# The Prevalence of Missing Incidents and Their Antecedents Among Older Adult MedicAlert Subscribers: Retrospective Descriptive Study

**DOI:** 10.2196/58205

**Published:** 2024-06-10

**Authors:** Antonio Miguel-Cruz, Hector Perez, Yoojin Choi, Emily Rutledge, Christine Daum, Lili Liu

**Affiliations:** 1 Department of Occupational Therapy Faculty of Rehabilitation Medicine University of Alberta Edmonton, AB Canada; 2 Faculty of Health University of Waterloo Waterloo, ON Canada; 3 Faculty of Medicine University of British Columbia Vancouver, BC Canada

**Keywords:** dementia, Alzheimer disease, memory loss, cognitive impairment, missing incident, wandering, critical wandering, older adults, retrospective design

## Abstract

**Background:**

With the population aging, the number of people living with dementia is expected to rise, which, in turn, is expected to lead to an increase in the prevalence of missing incidents due to critical wandering. However, the estimated prevalence of missing incidents due to dementia is inconclusive in some jurisdictions and overlooked in others.

**Objective:**

The aims of the study were to examine (1) the demographic, psychopathological, and environmental antecedents to missing incidents due to critical wandering among older adult MedicAlert Foundation Canada (hereinafter MedicAlert) subscribers; and (2) the characteristics and outcomes of the missing incidents.

**Methods:**

This study used a retrospective descriptive design. The sample included 434 older adult MedicAlert subscribers involved in 560 missing incidents between January 2015 and July 2021.

**Results:**

The sample was overrepresented by White older adults (329/425, 77.4%). MedicAlert subscribers reported missing were mostly female older adults (230/431, 53.4%), living in urban areas with at least 1 family member (277/433, 63.8%). Most of the MedicAlert subscribers (345/434, 79.5%) self-reported living with dementia. MedicAlert subscribers went missing most frequently from their private homes in the community (96/143, 67.1%), traveling on foot (248/270, 91.9%) and public transport (12/270, 4.4%), during the afternoon (262/560, 46.8%) and evening (174/560, 31.1%). Most were located by first responders (232/486, 47.7%) or Good Samaritans (224/486, 46.1%). Of the 560 missing incidents, 126 (22.5%) were repeated missing incidents. The mean time between missing incidents was 11 (SD 10.8) months. Finally, the majority of MedicAlert subscribers were returned home safely (453/500, 90.6%); and reports of harm, injuries (46/500, 9.2%), and death (1/500, 0.2%) were very low.

**Conclusions:**

This study provides the prevalence of missing incidents from 1 database source. The low frequency of missing incidents may not represent populations that are not White. Despite the low number of missing incidents, the 0.2% (1/500) of cases resulting in injuries or death are devastating experiences that may be mitigated through prevention strategies.

## Introduction

### Background

With the population aging, the number of people living with dementia will increase. Currently, approximately 55 million people have dementia globally [1]. With approximately 10 million new diagnoses each year, the total number of cases is expected to rise to 78 million by 2030 [1]. Canada is no exception to this trend, with at least 546,000 people currently living with dementia [2]. By 2030, the number of Canadians with dementia will reach at least 1,712,400 [3].

The disease burden cost associated with dementia is sizable in Canada. It was estimated that the direct costs (eg, long-term care) associated with dementia was CAD $10.4 billion (US $7.52 billion) in 2016, and it is expected to double by 2030 [2]. Half of the global cost of dementia is attributed to informal care (ie, family members and friends) [1]. It is estimated that, on average, care partners spend 26 to 35 hours per week caring for persons with dementia [1,4]. This overwhelming number of caregiving hours is attributed to personal care (ie, personal care such as bathing, feeding, and assisting with toileting) [5] and vigilance as a prevention strategy to prevent unattended exits, ultimately keeping persons living with dementia safe in their homes [6].

With increasing numbers of people living with dementia, the prevalence of missing incidents due to critical wandering is rising as well (refer to section *The Concept of Missingness and Critical Wandering and Its Risk Factors: A Brief Theoretical Background*). However, research on prevalence estimation on missing incidents due to critical wandering is inconclusive in some jurisdictions and overlooked in others. Limitations of the prior literature on this topic exist. First, there is a lack of consistency on reported prevalence [7], leading to disparate statistics; for example, McShane et al [8] reported that 40% of people with dementia become lost, and 5% do so repeatedly. The Alzheimer’s Association estimated that 60% of people with Alzheimer disease will wander [3], and a considerably larger set of studies showed that the prevalence of wandering varies from 11% to 60% [9,10]. Second, previous studies included low sample sizes from limited secondary data sources (eg, data not retained for >5 years) [11] such as police data and data obtained from newspaper report or social media [12-15], leading to a limited scope of the statistical analyses. Third, prevalence studies have been completed in the United States [16,17], Japan [18-20], and South Korea [21,22], leaving the prevalence of missing incidents among people with dementia in Canada largely unknown. This is an important gap in our knowledge because Canada has distinct social, health care, and geographic features as well as a harsh climate, making it challenging to extrapolate data from other countries for its unique context. As a result, the prevalence and risk factors of missing incidents due to critical wandering for Canadians living with dementia remain largely unknown.

### Objectives

The aims of this study were to examine (1) the demographic, psychopathological, and environmental antecedents to missing incidents due to critical wandering among older adult MedicAlert subscribers; and (2) the characteristics and outcomes of the missing incidents. We used a retrospective descriptive design. The sample included 434 older adult MedicAlert subscribers (hereinafter MedicAlert subscribers) involved in 560 missing incidents between January 2015 and July 2021.

### The Concept of Missingness and Critical Wandering and Its Risk Factors: a Brief Theoretical Background

Missingness is the phenomenon of going missing [11]. A missing person is an “individual that cannot be found” [23]. A missing person is “an individual whose whereabouts are unknown to members of their familial, social or professional networks where there is concern for either their own safety and wellbeing or that of others” [24]. A person can go missing intentionally or unintentionally. A person who goes missing unintentionally is said to be lost if the person is (1) “confused with current location in respect to finding other locations*”* and (2) “unable to reorient” [23]. In this research, we analyzed missing incidents related to persons (older adults) who go missing unintentionally. People living with dementia are at risk of unintentionally getting lost due to critical wandering. “Critical wandering” occurs when an individual living with dementia “leaves an institution or home [with or without the consent of their care partner] and is unaware of his or her situation in terms of place and/or time” [7]; the person is lost. Critical wandering is a necessary (but not a sufficient) condition for a missing incident to occur. A missing incident of a person living with dementia can occur when, for instance, this person is left unsupervised for a few minutes and has an episode of critical wandering [25]. Therefore, critical wandering and missing incident are 2 distinct concepts, although the literature in this field acknowledges that the former could lead to the latter [26].

Antecedents or risk factors influence the mechanisms preceding and contributing to missing incidents [11,26,27]. One way to classify antecedents is to determine whether characteristics are intrinsic (demographic and psychopathological or neurocognitive antecedents) or extrinsic (situational or environmental antecedents) to the missing individual. Demographic antecedents comprise sex, gender, age, and ethnicity of the missing individual. Psychopathological or neurocognitive antecedents are manifestations of behaviors related to cognitive or psychological impairment or mental illness, disorders, or distress. Finally, environmental antecedents may include social, cultural, political, economic, and weather conditions [11]. Another way to classify antecedents is to determine whether they are fixed or variable. A fixed antecedent is one that does not vary within individuals over time (eg, ethnicity). Conversely, a variable antecedent changes over time (eg, the age of an individual) [28]. Missing incidents can lead to consequences or outcomes for the missing person and their care partners [26]. For a missing person, these outcomes can range from returning home safely to minor injuries, major injuries, or even death [26].

### MedicAlert Service

MedicAlert is a Canadian-based service that can assist first responders and Good Samaritans in identifying an individual who has gone missing and connecting them with their care partners to help them return to their place of residence. The 2 primary tools used in the service are a medical ID object and a personal health information record. The ID object, which is typically in the form of a bracelet, contains key health conditions and a unique MedicAlert ID number specific to the individual. The MedicAlert ID number can then be used by authorized personnel to access a subscriber’s personal health information record, which contains extensive details about the subscriber’s medical conditions and medications as well as information on previous wandering history if provided by the subscriber or care partner. It is important to recognize that information is self-reported instead of provided by, or confirmed with, health providers, that is, a MedicAlert subscriber or care partners are at liberty to disclose details about the person’s situation and medical condition when the person goes missing. This information is relayed via a 24/7 hotline or by direct digital access by police dispatchers. When the missing person is found, the hotline operator or the police dispatcher notifies the care partners about the missing person’s location [29]. By linking care partners, first responders, and Good Samaritans, the goal is to safely return the missing person back home.

## Methods

### Ethical Considerations

The University of Waterloo ethics review board approved this study (43164).

### Study Design and Sample

We used a retrospective descriptive design. The sample included 434 older adult MedicAlert subscribers involved in 560 missing incidents between January 2015 and July 2021. Data were obtained from the MedicAlert subscriber and hotline call operators databases and summary notes made by hotline call operators. Both databases are linked through a unique MedicAlert subscriber ID number.

### Variables and Measures

We obtained information on the variables described in Textbox 1 (refer to Multimedia Appendix 1 for more details).

**Study variables.**

**Variable and description**

- Demographic antecedents: age, sex at birth, ethnic background, official Canadian languages spoken, province, and primary contact
- Psychopathological antecedents: medical conditions
- Environmental antecedents: domicile (urban vs rural) and living arrangement

Characteristics of the missing incidents: mode of mobility, the time of day and season in which the missing incident occurred, the family care partner’s involvement in response to the missing incident, who reported and found the missing person, point last seen or where the person was missing from, location in which the person was found (actual and self-reported), number of missing incidents, repeated missing incident history (actual and self-reported), mean time to the first missing incident (in days), mean time between missing incidents (in days), and survivability

### Procedures

#### Missing Incident Selection Procedure

Detailed information about the missing incidents were obtained from the summary notes made by the MedicAlert hotline operator when a call was received at the MedicAlert call center. These notes are documented by the operator each time a call is received and were in free-text format. We included missing incidents in which the MedicAlert subscribers (1) were aged ≥65 years, (2) went missing unintentionally, and (3) there was clear indication that the subscriber was actually lost (indications of disorientation or confusion or spatial navigation challenges). We excluded missing incidents that (1) were false positive reports (eg, GPS devices were activated and generated a record in the hotline access database, miscommunication between family members, and missing incident calls created for training purposes), (2) were a duplicate missing incident in which several follow-up calls were associated with the same missing incident, or (3) did not contain enough information from which to extract data.

Upon receiving the data set, 8 team members (including the authors) immersed themselves in the data set. Each read 60 different call summary notes and made notes on their contents in relation to the free-text fields in the MedicAlert subscriber database (eg, domicile [urban vs rural]; refer to Multimedia Appendix 1 for more details). Team members shared their observations during 2 subsequent meetings, and these were used to create a preliminary coding framework, including key definitions and the operationalization of each variable (refer to the next subsection for more details). Two team members, hereinafter referred to as analysts, screened, extracted, and coded relevant information from the summary notes made by the MedicAlert hotline operator using the coding framework. The analysts then completed a calibration exercise in which they independently applied the inclusion and exclusion criteria as well as coded 10 included missing incidents. The calibration exercise was conducted as follows: first, 10 cases were selected randomly; next, 2 researchers independently assessed the cases based on the inclusion and exclusion criteria; and, finally, the 2 researchers met and debriefed on the main causes of disagreements. In the calibration process, the team achieved a 90% agreement (ie, percentage of agreement calculated as the number of times a set of ratings is the same divided by the total number of units of observation that are rated). The analysts then screened and coded data for another 100 missing incidents (registries) independently and checked each other’s work. The coding framework was revised to improve the clarity of the definitions and the operationalization of the codes. The analysts met weekly to discuss missing incidents that were unclear or required a second opinion and revised the coding framework to increase clarity. When the coding framework was revised, the analysts reviewed the previously coded data against the revised coding framework and recoded as necessary. The analysts also sought feedback from the first author when conflicts arose in their screening and coding. In total, 7045 missing incidents were screened from the hotline access MedicAlert database; after applying the inclusion and exclusion criteria, 6485 (92.5%) incidents were excluded. The 6485 missing incidents were excluded due to false positive reports (n=5093, 78.84%), not enough information from which to extract data (n=1076, 16.66%), no indications of disorientation or confusion or spatial navigation challenges (n=270, 3.8%), and MedicAlert subscribers being aged <65 years (n=46, 0.65%).

#### Categories Generation and Operationalization of Variables

After the data set was cleaned, variables regarding antecedents to the missing incident were coded categorically based on previous research [30,31] and following Statistics Canada classifications whenever possible [32]. As some variables were stored in the form of free text, categories were generated inductively from the information contained in the free text (refer to the preceding subsection; eg, missing incident notes compiled by the hotline operator). Finally, all variables were operationalized as follows: dichotomous variables were coded as *0* or *1* (eg, MedicAlert subscriber’s sex), and each polytomous variable was represented by a set of binary variables, whose values codified each variable category.

### Data Analyses

We used descriptive statistics, including mean and SDs, to summarize the central values of distributions for continuous variables. We used the chi-square and Fisher exact—in the case of small, expected counts—tests for comparing categorical variables. Where appropriate, *t* tests (2-tailed) or the Mann-Whitney *U* test (2 independent groups, 2-tailed) and 1-way ANOVA (2-tailed) or the Kruskal-Wallis rank sum test (>2 groups, 2-tailed) were used for determining the difference between groups for continuous variables. Where appropriate, we used Cramer V and Pearson and Spearman ρ to determine correlations or associations between variables. As this was a retrospective descriptive study, each variable was examined separately [31]. Statistical analysis was conducted using SPSS software (version 28.0; IBM Corp). The α was set at .05.

## Results

### Demographic, Psychopathological, and Environmental Antecedents to Missing Incidents Among MedicAlert Subscribers

Table 1 shows the demographic and environmental antecedents to missing incidents among MedicAlert subscribers. Overall, 434 MedicAlert subscribers were involved in 560 missing incidents between January 2015 and July 2021. Regarding psychopathological or neurocognitive antecedents, in 79.5% (345/434) of the cases, MedicAlert subscribers self-reported living with dementia, and the remaining 20.5% (89/434) self-reported having other medical conditions, the most prevalent being short- and long-term memory loss and mental health issues such as depression, schizophrenia, and anxiety disorder. However, it is important to keep in mind that these data are self-disclosed at the time of subscribing to MedicAlert and thus may underestimate the true prevalence of dementia in this sample. The average age of the MedicAlert subscribers was 82.56 (SD 7.4) years, with a little more than half (230/431, 53.4%) identifying as female. The most prevalent age groups were 75 to 84 years (177/434, 40.8%) and 85 to 94 years (168/434, 38.7%), together representing 79.5% (345/434) of the sample. White older adults represented the vast majority (329/425, 77.4%) of the subscribers. In 55.8% (240/430) of the cases, the subscribers spoke English, with an additional 18.1% (78/430) who spoke another language or other languages in addition to English; notably, 11.6% (50/430) of the subscribers spoke neither of the 2 official Canadian languages, English and French. MedicAlert subscribers primarily resided in Ontario (199/341, 58.3%), British Columbia (57/341, 16.7%), or Quebec (50/341, 14.7%); and a vast majority (331/341, 97.1%) lived in urban areas. Living arrangements included with family (277/433, 64%) and in a facility (90/433, 20.8%), although 13.1% (57/433) reported living alone. Most of the subscribers (309/341, 90.6%) listed family members as their primary contact.

**Table 1:** Demographics and environmental antecedents of the sample (unit of analysis: MedicAlert subscriber).

Demographic characteristics		Persons without dementia and persons living with dementia	Persons without dementia	Persons living with dementia	Statistical tests (persons without dementia vs persons living with dementia)				*P* value	
					*F* test (*df*)		Chi-square (*df*)			
Age (y), mean (SD; range)		82.56 (7.4; 65-101)	83.90 (7.153; 66-101)	82.21 (7.381; 65-99)	0.93 (1,429)		—^a^		.33^b^	
**Sex assigned at birth (n=431), n (%)**						—		0.9 (1)		.33^c^
	Female	230 (53.4)	51 (11.8)	179 (41.5)						
	Male	201 (46.6)	37 (8.6)	164 (38.1)						
**Age group (y;** **n=434),** **n (%)**						—		7.8 (3)		.05^c^
	65-74	72 (16.6)	9 (2.1)	63 (14.5)						
	75-84	177 (40.8)	35 (8.1)	142 (32.7)						
	85-94	168 (38.7)	38 (8.8)	130 (29.9)						
	95-104	17 (3.9)	7 (1.6)	10 (2.3)						
	>105	0 (0)	0 (0)	0 (0)						
**Ethnic background (n=425), n (%)**						—		3.4 (3)		.34^c^
	Black^d^	23 (5.4)	6 (1.4)	17 (4)						
	Chinese	20 (4.7)	5 (1.2)	15 (3.5)						
	White	329 (77.4)	67 (15.8)	262 (61.6)						
	Other^e^	53 (14.3)	6(1.4)	47 (11.1)						
**Official Canadian languages spoken (n=430), n (%)**						—		6.4 (5)		.27^c^
	English only	240 (55.8)	47 (10.9)	193 (44.8)						
	English and other	78 (18.1)	11 (2.6)	67 (15.6)						
	Neither English nor French	50 (11.6)	10 (2.3)	40 (9.3)						
	French only	30 (7)	10 (2.3)	20 (4.7)						
	French and English	27 (6.3)	6 (1.4)	21 (4.9)						
	French and other	5 (1.2)	2 (0.5)	3 (0.7)						
**Province (n=341), n (%)**						—		6.6 (7)		.47^c^
	Ontario	199 (58.4)	36 (10.6)	163 (47.8)						
	British Columbia	57 (16.7)	11 (3.2)	46 (13.5)						
	Quebec	50 (14.7)	16 (4.7)	34 (9.9)						
	Alberta	18 (5.3)	3 (0.9)	15 (4.4)						
	Manitoba	9 (2.6)	2 (0.6)	7 (2.1)						
	Nova Scotia	4 (1.2)	0 (0)	4 (1.2)						
	Saskatchewan	3 (0.9)	1 (0.3)	2 (0.6)						
	New Brunswick	1 (0.3)	0 (0)	1 (0.3)						
	Newfoundland and Labrador	0 (0)	0 (0)	0 (0)						
	Prince Edward Island	0 (0)	0 (0)	0 (0)						
	Nunavut and Northwest Territories	0 (0)	0 (0)	0 (0)						
	Yukon	0 (0)	0 (0)	0 (0)						
**Population density (n=341), n (%)**						—		0.6 (1)		.44^c^
	Urban	331 (97.1)	66 (19.4)	265 (77.7)						
	Rural	10 (2.9)	3 (0.9)	7 (2.1)						
**Living arrangement (n=433), n (%)**						—		3.2 (3)		.36^c^
	With family	277 (64)	53 (12.2)	224 (51.7)						
	Facility	90 (20.8)	24 (5.5)	66 (15.2)						
	Alone	57 (13.1)	9 (2.1)	48 (11.1)						
	Other	9 (2.1)	2 (0.5)	7 (1.6)						
**Primary contact (n=341), n (%)**						—		3.2 (3)		.37^c^
	Family member	309 (90.6)	63 (18.5)	246 (72.1)						
	Other	16 (4.7)	5 (1.5)	11 (3.2)						
	Health and social care professionals	15 (4.4)	1 (0.3)	14 (4.1)						
	Staff of living facility	1 (0.3)	0 (0)	1 (0.3)						

^a^Not applicable.

^b^One-way ANOVA.

^c^Chi-square test.

^d^For example, African, Haitian, Jamaican, or Somali.

^e^Arab or West Asian (eg, Armenian, Egyptian, or Iranian), Latin American, South Asian, Korean, Mediterranean, Aboriginal (eg, Inuit, Métis, or American Indian), Filipino, Caribbean or West Indian (St Lucian or Antiguan), Southeast Asian, and Japanese.

Table 2 shows the history of missing incidents among MedicAlert subscribers. Subscribers self-reported no prior history of missing incidents at the time of subscribing to MedicAlert in 10.4% (45/433) of the cases, while 89.6% (388/433) disclosed having been involved in missing incidents repeatedly. Surprisingly, data from actual repeated missing incidents (ie, data that we accessed using the hotline access database) showed the opposite pattern: most of the subscribers went missing repeatedly in only 16.4% (71/434) of the cases. MedicAlert subscribers self-reported that the most common places to be found were outdoor public spaces (eg, highway or street; 184/308, 59.7%) or indoor public or communal spaces (eg, libraries; 85/308, 27.6%).

**Table 2:** Missing incidents history (unit of analysis: MedicAlert subscriber).

Missing incidents history		Persons without dementia and persons living with dementia, n (%)	Persons without dementia, n (%)	Persons living with dementia, n (%)	Statistical test (persons without dementia vs persons living with dementia), chi-square (*df*)		*P* value	
**Missing incident history (self-reported; n=433)**						0.9 (2)		.64
	None	45 (10.4)	7 (1.6)	38 (8.8)				
	Repeated (1^a^-4 times)	327 (75.5)	67 (15.5)	260 (60)				
	Habitual (>4 times)	61 (14.1)	14 (3.2)	47 (10.9)				
**Repeated missing incident history (actual; n=434)**						2.1 (1)		.14
	No	363 (83.6)	79 (18.2)	284 (65.4)				
	Yes	71 (16.4)	10 (2.3)	61 (14.1)				
**Possible locations for the missing person to be found (self-reported; n=308)**						4.4 (3)		.22
	Outdoor public space^b^	184 (59.7)	34 (11)	150 (48.7)				
	Indoor public or communal space^c^	85 (27.6)	18 (5.8)	67 (21.7)				
	Private home in the community^d^	32 (10.4)	11 (3.8)	21 (6.8)				
	Hospital (day program or day clinic)	7 (2.3)	1 (0.3)	6 (1.9)				

^a^After the first incident.

^b^Highway, street, alley, intersection, park, parking lot, outdoor bus stop, construction, or cemetery.

^c^Grocery store, shopping mall, train station, church, recreation center, library, physician’s office, bus, train, police station, or gas station.

^d^House, apartment, condominium, age ≥65 years condominium but without supportive living services.

The statistical tests in Tables 1 and 2 showed that MedicAlert subscribers with dementia and those without who were involved in missing incidents were similar with respect to mean age, ethnic background, official Canadian languages spoken, province of residence, population density, living arrangement, primary contact, possible location to be found during a missing incident (self-reported), missing incident history (self-reported), and repeated missing incident history (actual missing incidents); no statistically significant differences were found between the groups. MedicAlert subscribers with dementia and those without who were involved in missing incidents are significantly different (*χ*^2^_3_=7.8; *P*=.05) in terms of age groups. This result means that the prevalence of critical wandering was higher among older age groups of people with dementia, with the peak prevalence between ages 75 and 84 years declining somewhat among the older-aged adults.

### Characteristics of the Missing Incidents

#### Demographic and Psychopathological Antecedents

Missing incidents mostly involved people living with dementia (345/434, 79.6%) and those who were (1) female (230/431, 53.4%); (2) from the age groups 65-74 years (72/434, 16.6%), 75-84 years (177/434, 40.8%), and 85-94 years (168/434, 38.7%); (3) White (329/425, 77.4%); (4) English speaking (345/430, 80.2%); (5) living in an urban area (331/341, 97.1%); (6) mostly from Ontario (199/341, 58.4%), British Columbia (57/341, 16.7%), and Quebec (50/341, 14.7%); and (7) living with a family member (277/433, 64%) or in a facility (90/433, 20.8%).

#### Locations

Locations were analyzed in terms of point last seen or where the MedicAlert subscribers were missing from and located. MedicAlert subscribers were most frequently missing from private homes in the community (96/143, 67.1%) or residential living facilities (27/143, 18.9%); there were no statistical differences regarding the locations from which MedicAlert subscribers with dementia and those without went missing. Regarding the locations they were found, the most common places (313/382, 82%) were outdoor and indoor public spaces. Importantly, we found a statistically significant difference between point last seen or where the MedicAlert subscribers were missing from and where they were located (*χ*^2^_25_=42.3; *P*=.02; refer to Table 3 for more details). This result indicates that the MedicAlert subscribers were relatively active, with some degree of mobility. Even more interestingly, we found a moderate positive association between the possible locations to be found (self-reported) and the actual location where the person was found (Cramer V=0.213; *P*=.002).

**Table 3:** Characteristics of missing incidents. Point last seen or where the person was missing from and location in which the person was found (unit of analysis: missing incidents).

Characteristics of missing incidents (locations)			Persons without dementia and persons living with dementia, n (%)		Persons without dementia, n (%)		Persons living with dementia, n (%)		Statistical test (persons without dementia vs persons living with dementia), chi-square (*df*)			*P* value	
**Point last seen or where the person was missing from (n=143)**										3.4 (5)			.64
	Private home in the community	96 (67.1)		20 (14.8)		76 (51.7)							
	Residential living facility^a^	27 (18.9)		5 (4)		22 (15.4)							
	Indoor public space^b^	8 (5.6)		1 (0.7)		7 (4.7)							
	Other	5 (3.5)		2 (1.3)		3 (2.7)							
	Hospital (day program or day clinic)	5 (3.5)		0 (0)		5 (3.4)							
	Outdoor public space^c^	2 (1.4)		0 (0)		2 (1.3)							
**Location in which the person was found (n=382)**										10.2 (6)			.12
	Outdoor public space	202 (52.9)		42 (11.3)		160 (40.6)							
	Indoor public space	111 (29.1)		27 (7.8)		84 (22.6)							
	Private home in the community	40 (10.5)		5 (1.3)		35 (8.8)							
	Hospital (day program or day clinic)	12 (3.1)		2 (1)		10 (2.5)							
	Residential living facility	11 (2.9)		2 (0.5)		9 (8.8)							
	Other	4 (1.0)		3 (0.8)		1 (0.3)							
	Combination of >1 of the aforementioned locations	2 (0.5)		0 (0)		2 (0.5)							

^a^Long-term care center, assisted living facility, supportive living facility, lodge, or group home.

^b^Grocery store, shopping mall, train station, church, recreation center, library, or physician’s office.

^c^For example, highway, street, alley, intersection, park, or parking lot.

#### Mode of Mobility

While missing, the most common mode of traveling was on foot (248/270, 91.9%). The second most common mode of mobility was public transportation (12/270, 4.4%; refer to Table 4 for more details).

**Table 4:** Characteristics of missing incidents. Mode of mobility (unit of analysis: missing incidents; valid cases n=270).

Characteristics of missing incident			Persons without dementia and persons living with dementia, n (%)		Persons without dementia, n (%)		Persons living with dementia, n (%)		Statistical test (persons without dementia vs persons living with dementia), chi-square (*df*)			*P* value	
**Mode of mobility**										2.3 (6)			.89
	On foot^a^	248 (91.9)		57 (21.1)		191 (70.7)							
	Public transit^b^	12 (4.4)		3 (1.1)		9 (3.3)							
	Receiving a ride from someone else^c^	3 (1.1)		1 (0.4)		2 (0.7)							
	Driving own car	2 (0.7)		0 (0)		2 (0.7)							
	Other	2 (0.7)		0 (0)		2 (0.7)							
	Combination of several of the aforementioned modes	2 (0.7)		0 (0)		2 (0.7)							
	Long-range or transregional transit^d^	1 (0.4)		0 (0)		1 (0.4)							

^a^Walking.

^b^Bus, light rail transit, subway, or streetcar.

^c^Hitchhiking.

^d^Train, airplane, noncommuter bus, or ferry.

#### Temporality (Time and Seasonality)

Table 5 shows the temporality of missing incidents in terms of the time of day and season the person was reported missing. In general, missing incidents occurred mostly in the afternoon (262/560, 46.8%) and the evening (174/560, 31.1%), while the most common seasons for these incidents were summer and fall followed by spring (222/560, 39.6%; 154/560, 27.5%; and 113/560, 20.2%, respectively).

No statistical differences for the temporality variable by persons living with dementia and those without were found.

**Table 5:** Characteristics of missing incidents. Time and seasonality (unit of analysis: missing incidents; n=560).

Missing incident characteristics (time and seasonality)			Persons without dementia and persons living with dementia, n (%)		Persons without dementia, n (%)		Persons living with dementia, n (%)		Statistical test (persons without dementia vs persons living with dementia), chi-square (*df*)			*P* value	
**Time of day**										7.2 (2)			.03
	Afternoon (noon to 5:59 PM)	262 (46.8)		61 (11)		201 (35.1)							
	Evening (6 PM to 11:59 PM)	174 (31.1)		40 (7.1)		134 (23.3)							
	Morning (midnight to 11:59 AM)	124 (22.1)		15 (3.9)		109 (19.2)							
**Season**										3.3 (3)			.34
	Summer (June 1 to August 31)	222 (39.6)		47 (8.4)		175 (31.3)							
	Fall (September 1 to November 30)	154 (27.5)		34 (6.1)		120 (21.4)							
	Spring (March 1 to May 31)	113 (20.2)		17 (3)		96 (17.1)							
	Winter (December 1 to February 28)	71 (12.7)		18 (3.2)		53 (9.5)							

#### People Involved in the Missing Incident

Table 6 shows the people involved in the missing incidents in terms of the care partner involvement with MedicAlert in response to the missing incident. In the majority of cases (375/518, 72.4%), the family care partner had an involvement in response to the missing incident with MedicAlert, with no statistically significant difference found between the groups (people with dementia and those without). In 96.1% (467/486) of the cases, the MedicAlert subscribers who went missing were located by someone other than the care partner. In most of the cases, either first responders (232/486, 47.7%) or Good Samaritans (224/486, 46.1%) found the missing person. Again, no statistically significant difference was found between the groups involved in the missing incidents.

**Table 6:** Characteristics of missing incidents. People involved in the missing incident (unit of analysis: missing incidents).

Missing incident characteristics (people involved in the missing incident)			Persons without dementia and persons living with dementia, n (%)		Persons without dementia, n (%)		Persons living with dementia, n (%)		Statistical test (persons without dementia vs persons living with dementia), chi-square (*df*)			*P* value	
**Natural care partner involvement in response to incident with MedicAlert (n=518)**										0.4 (1)			.54
	Yes	375 (72.4)		80 (15.4)		295 (57)							
	No	143 (27.6)		27 (5.2)		116 (22.4)							
**Who reported and found the missing person (n=486)**										1.3 (3)			.72
	First responder^a^	232 (47.7)		54 (11.1)		178 (36.6)							
	Good Samaritan^b^	224 (46.1)		48 (9.9)		176 (36.2)							
	Family member or friend^c^	19 (3.9)		4 (0.8)		15 (3.1)							
	Other	11 (2.3)		1 (0.2)		10 (2.1)							

^a^Police, search and rescue member, fire department, or ambulance or paramedic.

^b^The Good Samaritan noticed that something was off with the missing person and called the hotline or was asked by the missing person to call the hotline; they were not formally involved in searching for the missing person.

^c^Informal care partner.

#### Outcomes of the Missing Incidents

Table 7 shows the outcomes of the missing incidents in terms of the number of missing incidents, repeated missing incidents, mean time to the first missing incident (in days), mean time between missing incidents (in days), and survivability. Overall, 22.5% (113/500) of the missing incidents were repeated missing incidents, with the mean number of missing incidents per MedicAlert subscriber being 1.290 (SD 0.914; range: 1-11). Moreover, the number of missing incidents per MedicAlert subscriber was almost the same for people living with dementia (mean 1.290, SD 0.801) and those without dementia (mean 1.300, SD 1.265). The mean time to the first missing incident (since registering with MedicAlert) was 343.8 (SD 376.2) days (mean 11, SD 11.3 months), whereas the mean time between missing incidents was shorter, that is, 328.0 (SD 366.6) days (mean 11, SD 10.8 months). This is expected because the mean time between missing incidents takes into account repeated missing incidents. In terms of survivability*,* only a small percentage of cases (46/500, 9%) involved people undergoing harm while missing; even more rare were missing incidents in which MedicAlert subscribers were found deceased (1/500, 0.2%). There was a trend toward adverse outcomes for MedicAlert subscribers living with dementia: they experienced increased repeated missing incidents and injuries (but these results were not statistically significant, *P*=.30), short mean time to the first missing incident (but these results were not statistically significant, *P*=.20), and short mean time between missing incidents (but these results were not statistically significant, *P*=.15). In other words, they went missing more frequently (1 missing incident every 317.08 days) than those subscribers who did not have dementia (1 missing incident every 370.41).

**Table 7:** Outcomes of the missing incidents.

Missing incident characteristics (outcomes)			Persons without dementia and persons living with dementia		Persons without dementia, n (%)		Persons living with dementia, n (%)		Statistical tests (persons without dementia vs persons living with dementia)											*P* value
									Mann-Whitney *U* test				*z* score			Chi-square (*df*)				
Missing incidents (n=434)			Mean 1.290 (SD 0.914; range 1-11)		1.300 (1.265)		1.290 (0.801)		14,412 (persons without dementia: n=89; persons with dementia: n=345)				−1.386			—^a^		.17^b^		
**Time (d; n=434), mean (SD)**																				
	MTFI^c^ (n=434)	Mean 343.79 (SD 376.20; range 6-2249)		374.82 (410.35)		335.79 (365.09)		14,000 (persons without dementia: n=89; persons with dementia: n=345)			−1.282			—			.20^b^			
	MTBI^d^ (n=434)	Mean 328.02 (SD 366.62; range 6-2249)		370.41 (411.34)		317.08 (354.00)		13,844 (persons without dementia: n=89; persons with dementia: n=345)			−1.429			—			.15^b^			
**Survivability (n=500), n (%)**										—		—			4.9 (4)				.30^e^	
	No apparent injuries or compromised health	453 (90.6)		94 (18.8)		359 (71.8)														
	Injuries or compromised health requiring emergency services and transfer to hospital	35 (7)		8 (1.6)		27 (5.4)														
	Minor injuries or health issues requiring some treatment at home^f^	10 (2)		1 (0.2)		9 (1.8)														
	Deceased	1 (0.2)		1 (0.2)		0 (0)														
	Injuries and concern for health requiring follow-up care^g^	1 (0.2)		0 (0)		1 (0.2)														

^a^Not applicable.

^b^Mann-Whitney *U* test.

^c^MTFI: mean time to the first missing incident.

^d^MTBI: mean time between missing incidents.

^e^Chi-square test.

^e^Getting *Band-Aids*, pain medications, cleaned up, and so on.

^f^Physician visit, walk-in clinic, and so on.

## Discussion

### Principal Findings

This retrospective descriptive study examined demographic, psychopathological, and environmental antecedents to missing incidents due to critical wandering among MedicAlert subscribers, as well as the characteristics and outcomes of these incidents. In doing so, we used a national registry of persons as a secondary data source of information (ie, the MedicAlert database). To date, much of the knowledge about missing individuals with dementia and those without is based on studies with small sample sizes that use social media and newspaper reports from the United States or elsewhere [7,11]. Thus, we aimed to address these limitations by using an extensive secondary data set. To our knowledge, this is the first study that has shed light on the phenomenon of missingness and critical wandering of individuals with dementia and those without in Canada. In addition, we were able to report the prevalence of repeated missing incidents, based on this database, an important figure that has been absent in previous studies.

The demographic characteristics of our study population showed that people involved in missing incidents were mostly older adults (345/434, 79.6%), female older adults (230/431, 53.4%), living in the most populated provinces in Canada (306/341, 89.7%), and living in urban areas with at least 1 family member (309/341, 90.6%). Importantly, the majority of MedicAlert subscribers (345/434, 79.5%) involved in missing incidents self-reported living with dementia. More interestingly, except for age group, we did not find statistically significant differences between the people living with dementia and those without with respect to demographic, psychopathological, and environmental antecedents to missing incidents due to critical wandering. In addition, MedicAlert subscribers were most frequently missing from private homes in the community (96/143, 67.1%), as expected; were found in a different place than where they were last seen (313/382, 82%; most commonly outdoor and indoor public spaces); and were traveling on foot (248/270, 91.9%) and by public transportation (12/270, 4.4%), and during the afternoon (262/560, 46.8%) and evening (174/560, 31.1%). Subscribers were located mostly by first responders (232/486, 47.7%) and Good Samaritans (224/486, 46.1%). In terms of outcomes, overall, MedicAlert subscribers were involved in 1 missing incident, with the mean duration between missing incidents being 11 (SD 10.8) months and the time elapsed between subscribing to MedicAlert and the first missing incident being 11 (SD 11.3) months. Finally, the vast majority of MedicAlert subscribers involved in missing incidents were returned home safely (453/500, 90.6%), reports of harm and injuries were very low (46/500, 9.2%), and death was a rare event (1/500, 0.2%).

We found that missing incidents involved mostly older adults (345/434, 79.6%), female older adults (230/431, 53.4%), and White older adults (329/425, 77.4%), with the majority living in urban areas in cities in Ontario, British Columbia, and Quebec provinces (306/341, 89.7%). These demographic results are consistent with previous studies [30,33,34] and can be explained by the fact that these groups were more prevalent in our sample and because our sample was not a representative sample. The higher prevalence of missing incidents among female older adults can be attributed to the higher prevalence of female older adults living with dementia because dementia typically affects people at a 2:1 female-to-male ratio [2]. As there is evidence that demographic characteristics may serve as risk factors for missingness [11,35,36], the next logical step is to determine whether the demographics variables we explored in this study are factors for missingness in this sample. In this study, we did not identify statistically significant differences between people living with dementia and those without with respect to all our variables. The most plausible explanation for this is that these 2 groups of people are essentially the same, that is, they have dementia and memory loss, a risk factor that can lead to critical wandering and, in turn, a missing incident. Another plausible explanation for the lack of between-group differences could be that the individuals (1) had dementia but did not disclose their medical condition at the time of first subscribing to MedicAlert, (2) had dementia but did not know about their diagnosis, or (3) did not have dementia at the time of registration but developed dementia over time or by the time they went missing.

We found that the majority of MedicAlert subscribers involved in missing incidents self-reported living with dementia (345/434, 79.5%). Importantly, among those who were involved in missing incidents but did not self-report living with dementia (89/434, 20.1%), memory loss was self-reported as a medical condition. Our result is aligned with previous studies that found that persons with mental or cognitive disabilities (eg, those with Alzheimer disease or dementia) are more prone to going missing [31,37,38]. The literature reports that neurocognitive deficits from dementia predispose individuals to missing incidents and contribute to the inability to independently return home. These neurocognitive deficits could include memory deficits, such as declarative memory (remembering facts and events), episodic memory (short-term memory for recent events and contexts), and visual agnosia (inability to recognize objects or places). In addition, executive function impairments and disease-related changes to visuospatial and subperceptual processing (especially in unfamiliar locations), which typically manifests as difficulty with navigation, can explain why an individual living with dementia cannot independently return home [26].

The prevalence of MedicAlert subscribers who repeatedly went missing was lower in the hotline database in comparison to repeated missing self-reported variables. This result was anticipated because previous studies suggest that care partners are reluctant to contact emergency services, such as 911 or programs to locate older adults who are having an episode of critical wandering and have gone missing [37]. As a self-reported variable, this result could be attributed to overreporting by care partners. However, more objective explanations can be given. First, care partners often initiate the search within their homes or places last seen, and because many persons with dementia are found near the place that they were last seen, either on their own property or in their own neighborhood, the care partner could locate the missing person before their decision to request assistance from external organizations [31,37]. Second, it is possible that care partners subscribed their family member into the MedicAlert program as a preventative measure. For individuals who repeatedly had episodes of critical wandering in the past, care partners could have implemented their own measures or interventions to avoid missing incidents, including MedicAlert subscriptions. The literature reports that these interventions include avoiding lapses of supervision, whether planned or unplanned, through the use of technology (eg, GPS) to monitor and locate missing older adults with dementia [39]. Finally, it might be possible that care partners chose not to use the MedicAlert hotline to locate missing individuals to avoid attention and stigma associated with a formal search if initiated. Numerous studies have reported that the uses of technologies and programs by people living with dementia and their care partners aiming to reduce the risks of getting lost have highlighted the importance of discreet technologies that are unnoticeable to reduce stigma [40,41].

Our study paves the way for new services and interventions that can be offered by MedicAlert. The services may include implementing preventative strategies to decrease the risk of going missing through threshold alerts in mobile phone apps. According to the literature, a leading feature being implemented in mobile phone apps were alert systems, such as wandering alerts [42]. These apps could send threshold alerts or reminders to care partners when the mean time between missing incidents and the mean time to the first missing incident for a particular MedicAlert subscribers is approaching. The same can be true for common months or the time of day that MedicAlert subscribers tend to go missing. As many MedicAlert subscribers were located mostly by first responders and Good Samaritans, another option to explore is the use of a mobile alert app to engage community volunteers to help locate missing persons with dementia. Community ASAP, a mobile alert system that engages community citizens as volunteers to look for missing persons with dementia, has demonstrated to be an accurate and useful app [43].

For MedicAlert subscribers involved in missing incidents, many were returned home safely (453/500, 90.6%), with few reported harms or injuries (46/500, 9.2%), and death was rare (1/500, 0.2%). Regarding mortality rates when a person with dementia goes missing, the literature shows high variability (between 0.7% and 32%) [44,45]. In this study, the low reports of harm and death can be explained mainly by 2 factors. First, the environmental conditions at the time the MedicAlert subscribers went missing were favorable: subscribers went missing in urban areas while traveling on foot (248/270, 91.9%) or using public transportation (12/270, 4.4%) during the day (262/560, 46.8%) in the warmest months of the year (eg, low chance of severe weather; 358/560, 63.9%). The literature reports that the causes of high mortality rates in people with dementia who go missing include severe weather; driving; and walking near roadways, bodies of water, or in isolated areas [46,47]. These scenarios were very different from what we found in our study. Second, we found that in a high proportion of missing incidents (504/560, 90%), the MedicAlert subscribers were wearing their ID bracelet. We could intuitively affirm that the MedicAlert program prevents injuries and saves lives, but this affirmation would have to be demonstrated in a formal study. Therefore, a next logical step would be to conduct a study to determine whether the MedicAlert program addresses the problem for which it was designed, that is, to help those who are having an episode of critical wandering to return home safely. Our study also shows what some investigators have determined regarding the potential interrelatedness of risk factors for going missing [48]. While most of the outcomes during missing incidents were positive (death was rare), the complex interplay of demographic, psychopathological, and environmental antecedents of MedicAlert subscribers need further exploration.

### Study Limitations

Our study has some limitations. Limitations were posed by the MedicAlert data set itself. First, missing incidents are also captured in data held by first responders (police, search and rescue organizations, paramedics, etc), and, because MedicAlert data are subscription-based data, there are inherent self-selection biases. Second, while inquiring about the data entry process at the MedicAlert subscribers’ level, we discovered that a high percentage of data were stored raw (free-text fields) and not in analysis-ready format. Consequently, the available information did not allow us to categorize our data with the desired level of granularity. Third, the self-reported nature of the data caused missing data in some variables (eg, the use of de-escalation techniques to avoid missing incidents, whether a MedicAlert subscriber has special needs, and what constitutes a trigger for a missing incident). As the percentage of missing data in these variables was large (ie, >40%), we excluded them from the analyses as recommended in the literature [49]. The missing data will not allow for further comprehensive statistical analysis for these unmeasured confounding variables. Fourth, the database lacked some important outcome variables; for example, we were unable to determine for how long MedicAlert subscribers went missing, the response time (ie, time elapsed between the call to the hotline and the arrival of the first responders to assist a missing person) or the turnaround time of the missing incidents (ie, the time it takes to return a missing person to their residence). In summary, because this study used a secondary data source that had not been compiled for research purposes, we faced the same common limitations reported in other studies that use this kind of data source [50-52]. Notwithstanding these limitations, we believe that the results obtained in this study are very valuable for partially understanding the phenomenon of older adults with dementia and memory loss going missing in Canada. The data set used in this study represents a small portion of people living with dementia in Canada; by virtue of it being a paid subscription service, not everyone uses it. In future research, other sources of data also need to be considered (police and search and rescue data) to get a fuller picture of the prevalence of persons living with dementia who go missing.

### Conclusions

In the data set used, missing incidents involved mostly female older adults living with dementia from an urban area (331/341, 97.1%). Overall, the majority of MedicAlert subscribers involved in missing incidents returned home safely (453/500, 90.6%). However, 9.2% (46/500) of the cases resulted in some form of minor or serious injuries and death. Of the 560 missing incidents, 126 (22.5%) were repeated missing incidents. This paves the way to more accurately describe the prevalence of missing incidents and their consequences and outcomes so that we can develop targeted intervention strategies to prevent missing incidents or locate missing persons.

## Appendix

Multimedia Appendix 1 Definitions of variables and measures.

## References

[1] (2023). Dementia. World Health Organization.

[2] Chambers LW, Bancej C, Mcdowell I (2016). Prevalence and monetary costs of dementia in Canada: population health expert panel. The Alzheimer Society of Canada and Public Health Agency of Canada.

[3] (2022). Navigating the path forward for dementia in Canada. Alzheimer Society is Canada.

[4] Unpaid caregiver challenges and supports. Canadian Institute for Health Information.

[5] Brodaty H, Donkin M (2009). Family caregivers of people with dementia. Dialogues Clin Neurosci.

[6] Houston AM, Brown LM, Rowe MA, Barnett SD (2011). The informal caregivers' perception of wandering. Am J Alzheimers Dis Other Demen.

[7] Petonito G, Muschert GW, Carr DC, Kinney JM, Robbins EJ, Brown JS (2013). Programs to locate missing and critically wandering elders: a critical review and a call for multiphasic evaluation. Gerontologist.

[8] McShane R, Gedling K, Kenward B, Kenward R, Hope T, Jacoby R (1998). The feasibility of electronic tracking devices in dementia: a telephone survey and case series. Int J Geriatr Psychiatry.

[9] Teri L, Larson EB, Reifler BV (1988). Behavioral disturbance in dementia of the Alzheimer's type. J Am Geriatr Soc.

[10] Hope T, Tilling KM, Gedling K, Keene JM, Cooper SD, Fairburn CG (2004). The structure of wandering in dementia. Int J Geriat Psychiatry.

[11] Ferguson L (2022). Risk factors and missing persons: advancing an understanding of ‘risk’. Humanit Soc Sci Commun.

[12] Schonfeld L, King-Kallimanis B, Brown LM, Davis DM, Kearns WD, Molinari VA, Werner DH, Beattie ER, Nelson AL (2007). Wanderers with cognitive impairment in Department of Veterans Affairs nursing home care units. J Am Geriatr Soc.

[13] King-Kallimanis B, Schonfeld L, Molinari VA, Algase D, Brown LM, Kearns WD, Davis DM, Werner DH, Beattie ER, Nelson AL (2010). Longitudinal investigation of wandering behavior in Department of Veterans Affairs nursing home care units. Int J Geriatr Psychiatry.

[14] Kiely DK, Morris JN, Algase DL (2000). Resident characteristics associated with wandering in nursing homes. Int J Geriatr Psychiatry.

[15] Volicer L, van der Steen JT, Frijters DH (2013). Involvement in activities and wandering in nursing home residents with cognitive impairment. Alzheimer Dis Assoc Disord.

[16] Algase DL, Antonakos C, Beattie ER, Beel-Bates CA, Yao L (2009). Empirical derivation and validation of a wandering typology. J Am Geriatr Soc.

[17] Algase DL, Beattie ER, Antonakos C, Beel-Bates CA, Yao L (2010). Wandering and the physical environment. Am J Alzheimers Dis Other Demen.

[18] Kikuchi K, Ijuin M, Awata S, Suzuki T (2016). [A study on the mortality patterns of missing and deceased persons with dementia who died due to wandering]. Nihon Ronen Igakkai Zasshi.

[19] Murata S, Takegami M, Onozuka D, Nakaoku Y, Hagihara A, Nishimura K (2021). Incidence and mortality of dementia-related missing and their associated factors: an ecological study in Japan. J Epidemiol.

[20] Okita M, Hanyu H, Hirao K, Shimizu S, Umahara T, Sakurai H (2016). Missing incidents in individuals with dementia attending a memory clinic. J Am Geriatr Soc.

[21] Hong GR, Song JA (2009). Relationship between familiar environment and wandering behaviour among Korean elders with dementia. J Clin Nurs.

[22] Jeong JG, Song JA, Park KW (2016). A relationship between depression and wandering in community-dwelling elders with dementia. Dement Neurocogn Disord.

[23] Koester RJ (2008). Lost Person Behavior: A Search and Rescue Guide on Where to Look - For Land, Air and Water.

[24] Taylor C, Woolnough PS, Dickens GL (2018). Adult missing persons: a concept analysis. Psychol Crime Law.

[25] Puthusseryppady V, Coughlan G, Patel M, Hornberger M (2019). Geospatial analysis of environmental risk factors for missing dementia patients. J Alzheimers Dis.

[26] Rowe M, Houston A, Molinari V, Bulat T, Bowen ME, Spring H, Mutolo S, McKenzie B (2015). The concept of missing incidents in persons with dementia. Healthcare (Basel).

[27] Neubauer NA, Liu L (2021). Development and validation of a conceptual model and strategy adoption guidelines for persons with dementia at risk of getting lost. Dementia (London).

[28] Kraemer HC, Kazdin AE, Offord DR, Kessler RC, Jensen PS, Kupfer DJ (1997). Coming to terms with the terms of risk. Arch Gen Psychiatry.

[29] Medic-Alert Foundation Canada homepage. Medic-Alert Foundation Canada.

[30] Rowe MA, Greenblum CA, Boltz M, Galvin JE (2012). Missing drivers with dementia: antecedents and recovery. J Am Geriatr Soc.

[31] Bantry White E, Montgomery P (2015). Dementia, walking outdoors and getting lost: incidence, risk factors and consequences from dementia-related police missing-person reports. Aging Ment Health.

[32] Classifications, variables and statistical units. Statistics Canada.

[33] Tu MC, Pai MC (2006). Getting lost for the first time in patients with Alzheimer's disease. Int Psychogeriatr.

[34] Lissemore FM, Shatzman S, Clark N, Nash J, Miller R, Lerner AJ (2019). Dementia reported missing: use of an online search engine to track outcomes in persons with dementia reported missing. Alzheimer Dis Assoc Disord.

[35] Cohen IM, McCormick AV, Plecas D A review of the nature and extent of uncleared missing persons cases in British Columbia. University College of the Fraser Valley.

[36] Cohen IM, Plecas D, McCormick AV (2009). A comparison of aboriginal and non-aboriginal missing persons in British Columbia where foul play has not been ruled out. University College of the Fraser Valley.

[37] Bowen ME, McKenzie B, Steis M, Rowe M (2011). Prevalence of and antecedents to dementia-related missing incidents in the community. Dement Geriatr Cogn Disord.

[38] Holmes L, Shalev-Greene K, Clarke C, Pakes F (2015). The impact of living with missing incidents: how the experience and fear of missing incidents affects people with dementia and those who care for them. Proceedings of the 25th Alzheimer Europe Conference: Dementia: Putting Strategies and Research Into Practice.

[39] Neubauer NA, Lapierre N, Ríos-Rincón A, Miguel-Cruz A, Rousseau J, Liu L (2018). What do we know about technologies for dementia-related wandering? A scoping review: examen de la portée : que savons-nous à propos des technologies de gestion de l'errance liée à la démence?. Can J Occup Ther.

[40] Neubauer N, Spenrath C, Philip S, Daum C, Liu L, Miguel-Cruz A (2022). Identifying adoption and usability factors of locator devices for persons living with dementia. Dementia (London).

[41] Peeters MW, Schouten G, Wouters EJ (2021). Wearables for residents of nursing homes with dementia and challenging behaviour: values, attitudes, and needs. Gerontechnology.

[42] Guo Y, Yang F, Hu F, Li W, Ruggiano N, Lee HY (2020). Existing mobile phone apps for self-care management of people with Alzheimer disease and related dementias: systematic analysis. JMIR Aging.

[43] Neubauer N, Daum C, Miguel-Cruz A, Liu L (2021). Mobile alert app to engage community volunteers to help locate missing persons with dementia. PLoS One.

[44] Woolford MH, Weller C, Ibrahim JE (2017). Unexplained absences and risk of death and injury among nursing home residents: a systematic review. J Am Med Dir Assoc.

[45] Rowe MA, Bennett V (2003). A look at deaths occurring in persons with dementia lost in the community. Am J Alzheimers Dis Other Demen.

[46] Kibayashi K, Shojo H (2003). Accidental fatal hypothermia in elderly people with Alzheimer's disease. Med Sci Law.

[47] Hunt LA, Brown AE, Gilman IP (2010). Drivers with dementia and outcomes of becoming lost while driving. Am J Occup Ther.

[48] Kiepal LC, Carrington PJ, Dawson M (2012). Missing persons and social exclusion. Can J Sociol.

[49] Jakobsen JC, Gluud C, Wetterslev J, Winkel P (2017). When and how should multiple imputation be used for handling missing data in randomised clinical trials - a practical guide with flowcharts. BMC Med Res Methodol.

[50] Johnson EK, Nelson CP (2013). Values and pitfalls of the use of administrative databases for outcomes assessment. J Urol.

[51] Hubbard SL, Fitzgerald SG, Reker DM, Boninger ML, Cooper RA, Kazis LE (2006). Demographic characteristics of veterans who received wheelchairs and scooters from Veterans Health Administration. J Rehabil Res Dev.

[52] Rios A, Miguel Cruz A, Guarín MR, Caycedo Villarraga PS (2014). What factors are associated with the provision of assistive technologies: the Bogotá D.C. case. Disabil Rehabil Assist Technol.

