# Supplementary material for: The Prevalence of Missing Incidents and Their Antecedents Among Older Adult MedicAlert Subscribers: Retrospective Descriptive Study
Source: JMIR Aging. 2024 Jun 10;7:e58205. doi: 10.2196/58205 (PMC11196911; doi:10.2196/58205)
Supplement: Multimedia Appendix 1 [file aging_v7i1e58205_app1.pdf]

Supplemental Material

Table A. 1. Variables and measures definition

Category	Subcategory	Variable name	Variable type and Definition	Source/format
Antecedents	Demographic	Age	Continuous. Defined as to the age of a person of interest at last birthday (or relative to a specified, well-defined reference date)[a].	MA[b]/Structured[c]
		Sex at birth	Categorical. We followed Statistics Canada's main classification of sex at birth[d].	MA[a]/Structured[b]
		Ethnic background	Categorical. Refers to the ethnic or cultural origins of the person's ancestors[e]. Also known as population group	MA[a]/Structured[b]
		Canadian official languages spoken	Categorical. Refers to whether the person can conduct a conversation in English only, French only, in both or in neither language[f].	MA[a]/Structured[b]
		Province	Categorical. The Canadian province of residency at the moment they registered at the MedicAlert®[g]	MA[a]/Free text[h]
		Primary contact	Categorical.	MA[a]/Structured[b]
	Psychopathological	Medical condition (self-reported)	Categorical. A communication or relationship between people that is characterized by intimacy and personal familiarity.	MA[a]/Structured[b]
	Environmental	Population Density	Categorical. Population per unit area where the person lives	MA[a]/Free text[h]
		Living arrangement	Categorical. Refers to with whom the person involved in the missing incident lives with	MA[a]/Structured[b]
Missing incidents	Characteristics	Disorientation and confusion and spatial navigation issues	Categorical. Whether the person involved in the missing incident had disorientation and confusion and spatial navigation issues	HL[i]/Free text[h]
		Locations. Point last seen or where the person is missing from	Categorical. Location missing person was last seen or heard from	HL[i]/Free text[h]
		Location in which the person was found	Categorical. Place in which the person was actually found (self-reported)	HL[i]/Free text[h]
		Possible locations to be found (self-reported)	Categorical. Place in which the person was found (self-reported)	HL[i]/Free text[h]
		Mode of mobility	Categorical. Refer to the mode of mobility used by the missing person	HL[i]/Free text[h]
		Time of the day	Categorical. This is the time the incident was notified to the MedicAlert® hotline	HL[i]/ Structured[b]
		Season	Categorical. Predominate season during the incident	HL[i]/Free text[h]
		Natural caregiver involvement in response to incident	Categorical. Whether the caregiver was involved in locating the missing person	HL[i]/Free text[h]
		Who reported and found the person	Categorical. Reported missing by a third party	HL[i]/Free text[h]
		Missing incident history (self-reported)	Categorical. Whether MedicAlert® ´s subscriber self reported that was involved in more than one missing incident in the period analyzed	MA[a] /Free text[h]
	Outcomes	Number of missing incidents	Continuous. Total number of missing incidents per MedicAlert® ´s subscriber	HL[b]/Structured[c]
		Repeated missing incident history (actual)	Categorical. Whether MedicAlert® ´s subscriber was involved in more than one missing incident in the period analyzed	HL[i]/ Structured[b]

		Time to the first missing incident (MTFI) (in days)	Continuous<br>Calculated as the time elapsed from date to subscribers subscribed into MedicAlert® ´s database to the date of the first missing incident occurred (hotline access)	HL[i]/derived
		Mean time between missing incidents (MTBI) (in days)	Continuous<br>Calculated as the average time between all missing incidents.	HL[i]/derived
		Survivability	Categorical. Whether the person reported in the missing incident was found alive but injured of any kind	MA[a] /Free text[h]

Notes

a. When categorized, classification of age group followed Statistics Canada. Available at: https://www23.statcan.gc.ca/imdb/p3VD.pl?Function=getVD&TVD=252430
b. MedicAlert® ´s subscribers data base
c. Data or variable is stored in a structured manner in the data base. No further pre processing of the variable is needed
d. Available at https://www23.statcan.gc.ca/imdb/p3VD.pl?Function=getVD&TVD=252430
e. Classification of Ethnic background. October 01, 2021 to current. Statistics Canada. Available at: https://www23.statcan.gc.ca/imdb/p3VD.pl?Function=getVD&TVD=402936
f. Classification of Canadian Knowledge of Official Languages. October 01, 2021 to current available at https://www23.statcan.gc.ca/imdb/p3VD.pl?Function=getVD&TVD=281265
g. Classification of Canadian Provinces. Available at https://www.statcan.gc.ca/en/reference/province
h. Data need to be derived or processed following qualitative data analyses approach.
i. MedicAlert® hotline access data base
